# GATA6 Is a Crucial Regulator of Shh in the Limb Bud

**DOI:** 10.1371/journal.pgen.1004072

**Published:** 2014-01-09

**Authors:** Elena Kozhemyakina, Andreia Ionescu, Andrew B. Lassar

**Affiliations:** Department of Biological Chemistry and Molecular Pharmacology, Harvard Medical School, Boston, Massachusetts, United States of America; University of Oxford, United Kingdom

## Abstract

In the limb bud, patterning along the anterior-posterior (A-P) axis is controlled by Sonic Hedgehog (Shh), a signaling molecule secreted by the “Zone of Polarizing Activity”, an organizer tissue located in the posterior margin of the limb bud. We have found that the transcription factors GATA4 and GATA6, which are key regulators of cell identity, are expressed in an anterior to posterior gradient in the early limb bud, raising the possibility that GATA transcription factors may play an additional role in patterning this tissue. While both GATA4 and GATA6 are expressed in an A-P gradient in the forelimb buds, the hindlimb buds principally express GATA6 in an A-P gradient. Thus, to specifically examine the role of GATA6 in limb patterning we generated *Prx1-Cre; GATA6^fl/fl^* mice, which conditionally delete GATA6 from their developing limb buds. We found that these animals display ectopic expression of both Shh and its transcriptional targets specifically in the anterior mesenchyme of the hindlimb buds. Loss of *GATA6* in the developing limbs results in the formation of preaxial polydactyly in the hindlimbs. Conversely, forced expression of GATA6 throughout the limb bud represses expression of Shh and results in hypomorphic limbs. We have found that GATA6 can bind to chromatin (isolated from limb buds) encoding either *Shh* or *Gli1* regulatory elements that drive expression of these genes in this tissue, and demonstrated that GATA6 works synergistically with FOG co-factors to repress expression of luciferase reporters driven by these sequences. Most significantly, we have found that conditional loss of Shh in limb buds lacking GATA6 prevents development of hindlimb polydactyly in these compound mutant embryos, indicating that GATA6 expression in the anterior region of the limb bud blocks hindlimb polydactyly by repressing ectopic expression of Shh.

## Author Summary

Sonic Hedgehog (Shh) is a crucial regulator of the growth and anterior-posterior patterning of the developing limb bud, and is produced in the “Zone of Polarizing Activity” in the posterior of the limb bud. Here, we demonstrate that GATA4 and GATA6 (members of the GATA family of transcription factors) are expressed in the anterior mesenchyme of mouse limb buds and that limb bud-specific deletion of GATA6 results in ectopic expression of Shh and its target genes (such as Gli1) in the anterior limb bud mesenchyme, resulting in preaxial polydactyly. Conversely, over-expression of GATA6 in limb buds causes down-regulation of Shh and its target genes, resulting in a decreased number of digits. We also show that GATA6 binds to the sequences that regulate expression of either *Shh* or *Gli1*, and that simultaneous deletion of both *GATA6* and *Shh* genes in developing limb buds rescues the polydactylous hindlimb phenotype of GATA6 mutants. Our findings indicate that GATA6 is necessary to repress ectopic expression of both Shh and hedgehog transcriptional targets in the anterior region of the mouse hindlimb bud, and thus demonstrate that GATA transcription factors, in addition to being regulators of cell identity, are important negative regulators of ectopic Shh expression in the limb bud.

## Introduction

In the limb bud, patterning along the anterior-posterior (A-P) axis (i.e., thumb to little finger) is controlled by the “Zone of Polarizing Activity” or ZPA, an organizer tissue located in the posterior margin of the limb bud. Sonic Hedgehog (Shh) is a signaling molecule that is produced by the ZPA and is essential for both correct growth and patterning of the vertebrate limb [1], [2]. Deletion of *Shh* from the mouse genome results in a severe phenotype with loss of all digits in the fore- and hindlimbs except for one, most anterior digit [3]. Shh produced by the ZPA maintains the expression of Fibroblast Growth Factor (FGF) family members in the Apical Ectodermal Ridge (AER), an epithelial signaling center running along the distal tip of the limb bud (for review, see [4]). It does so by inducing the expression of Grem1 in the limb bud mesenchyme, which in turn antagonizes BMP signaling, a repressor of FGF expression in the AER (reviewed in [4], [5]). A spatial gradient of Shh, with the highest level of Shh in the posterior region of the limb bud mesenchyme and the lowest in the more anterior region, is absolutely required for correct patterning of the limbs along the anterior-posterior axis.

In vertebrates, Hedgehog signals are transduced by Gli1, Gli2, and Gli3 transcription factors (reviewed in [6]). In the absence of Hedgehog signaling, Gli3-full length (FL) (which is a weak transcriptional activator) is efficiently processed by ubiquitin-dependent proteolysis to give rise to a potent transcriptional repressor termed Gli3R [7]. In addition, in the absence of hedgehog signals, Gli2-full length (FL) (which is a weak transcriptional activator) has a short half-life, also due to ubiquitin-dependent proteolysis [8], [9]. When hedgehog ligands bind to the trans-membrane proteins Patched1/2 (Ptc1/2), it results in the translocation of the trans-membrane protein smoothened to the base of the primary cilium [10]. The localization of smoothened to the primary cilium blocks the production of Gli3R, and results in the stabilization of both Gli3FL and Gli2FL [11]. In the absence of Gli3R production, Gli3FL and Gli2FL induce the expression of hedgehog-responsive genes, which include Gli1.

The high posterior to low anterior gradient of Shh in the limb bud creates a spatial gradient of Gli3R, with the lowest levels of Gli3R in the posterior limb bud mesenchyme (where Shh signaling is high) and the highest levels of Gli3R in the anterior limb bud (where Shh signaling is low; reviewed in [5]). This gradient of Gli3R production in the limb is critical for specification of the proper number and pattern of digits. *Gli3* ^−/−^ mice display polydactyly (i.e., excess digits; [12], [13]), as do mice engineered to express only a mutant form of Gli3FL that cannot be processed to give rise to Gli3R (*Gli3* ^P1-4/P1-4^ mice; [14]). In both *Gli3* ^−/−^ and *Gli3* ^P1-4/P1-4^ mice, the expression of Shh and Grem1 is aberrantly induced in the anterior region of the limb bud, resulting in the anterior expansion of FGF4 expression in the AER [12], [13], [14]. Thus, Gli3R production in the anterior region of the limb bud is necessary to block aberrant expression of both Shh and Grem1 in the anterior mesenchyme of the limb buds and associated anterior expansion of FGF4 expression in the AER. A consequence of the ectopic expression of Shh in the anterior mesenchyme of the limb buds and subsequent anterior expansion of FGF4 expression in the AER is polydactyly [12], [13], [14].

Preaxial polydactyly (a type of polydactyly with supernumerary digits forming in the anterior limb region) is a frequently occurring limb abnormality in which limb asymmetry is disrupted during development due to formation of an ectopic ZPA in the anterior of the limb bud. Preaxial polydactyly in mice and humans has a number of genetic causes, with one of the common being mutations in the long-range, limb-specific enhancer of the *Shh* gene, causing ectopic Shh expression in the anterior limb bud mesenchyme [15], [16], [17], [18]. In addition, decreased levels of either of the transcription factors Twist1 or Alx4, both of which are expressed in the anterior limb bud mesenchyme, where they repress ectopic expression of Shh in this region [19], [20], [21], [22], can induce preaxial polydactyly. Increased levels of the transcription factor Hand2, which is known to promote expression of Shh in the posterior limb bud mesenchyme, can also cause polydactyly [23], [24].

In this study, we have noted that both GATA4 and GATA6 are expressed in an anterior-posterior (A-P) gradient in the forelimb buds (i.e., high anterior/low posterior), and that hindlimb buds principally express GATA6 in an A-P gradient. These expression patterns raised the possibility that GATA4 and GATA6 may play a critical role in establishing the anterior-posterior morphogenetic field within the developing limb bud. In support of this notion we have found that conditional deletion of *GATA6* from mouse limb buds results in ectopic expression of *Shh* and its target genes specifically in the anterior mesenchyme of the hindlimb buds, causing hindlimb preaxial polydactyly. Conversely, transgenic over-expression of GATA6 in both fore- and hindlimb buds represses expression of both Shh and its target genes, resulting in A-P patterning defects and hypomorphic limbs. We have identified conserved GATA6 binding sites in the regulatory regions of both the *Gli1* and *Shh* genes, which interact with GATA6 in limb bud tissue as assayed by chromatin IP. Furthermore, we have found that GATA6 can repress the expression of reporter genes driven by these sequences. Importantly, we have found that conditional loss of Shh in limb buds lacking GATA6 prevents formation of hindlimb polydactyly in these compound mutant embryos, indicating that GATA6 expression in the anterior region of the limb bud blocks hindlimb polydactyly by repressing ectopic expression of Shh. Together, our findings indicate that GATA6 is another crucial transcriptional regulator of Shh signaling in the limb bud.

## Results

### GATA4 and GATA6 are differentially expressed in the anterior mesenchyme of the developing limb buds and GATA6 loss results in hindlimb preaxial polydactyly

**Figure 1:**
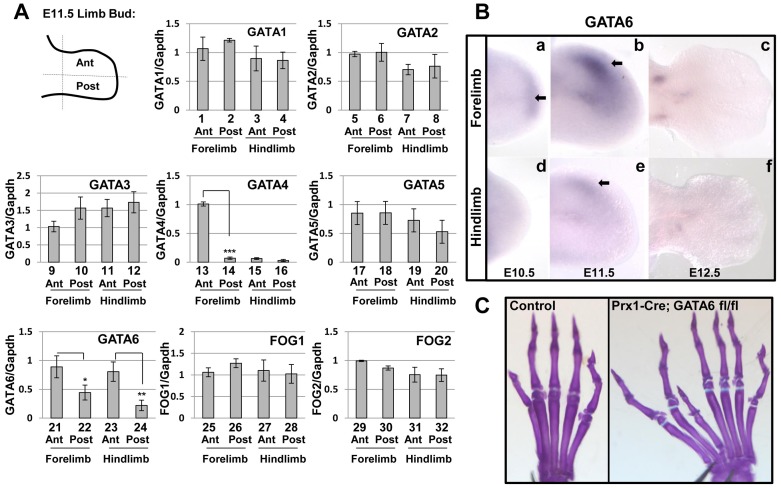
GATA and FOG factors are expressed in the limb bud mesenchyme, loss of GATA6 from limb bud mesenchyme results in hindlimb preaxial polydactyly. (**A**) Expression of GATA1-6 and FOG1/2 factors relative to GAPDH was assayed by RT-qPCR in anterior (Ant) or posterior (Post) regions of E11.5 mouse forelimbs or hindlimbs. *p<0.05, **p<0.01, ***p<0.001; Student's test. Error bar indicates standard deviation, n = 3. In each case, the transcript abundance (relative to GAPDH) in the anterior forelimb of the first embryo examined (out of 3) was set to equal 1.0; the transcript abundance (relative to GAPDH) in either other regions of this embryo or in other embryos was normalized to this arbitrary set point. (**B**) Whole mount in situ hybridization analysis of GATA6 expression in E10.5 (a, d), E11.5 (b, e), and E12.5 (c, f) WT forelimbs (a–c) or hindlimbs (d–f) performed with a 3′ UTR mouse GATA6 probe [28]. Arrows point to the location of GATA6 expression. At least 5 embryos of each stage were analyzed and representative samples are shown. (**C**) Alcian Blue/Alizarin Red staining of the hindlimb of either a P21 control mouse (left) or a *Prx1*-Cre; *GATA6* ^fl/fl^ mouse (right) is displayed. At least 20 animals of *Prx1*-Cre; *GATA6* ^fl/fl^ genotype were analyzed and preaxial polydactyly was observed with 100% penetrance in these mutants (representative animal with 7 hindlimb digits is shown in the right panel). Anterior digits are on the left side of each image.

Prior work has found that expression of GATA3, GATA5, and GATA6 can be detected in mouse limb buds using RT-qPCR analysis, and demonstrated that the latter two GATA factors are transiently expressed in a gradient (high in proximal and low in distal regions) in the developing limb bud [25]. Subsequent analysis has revealed that GATA6 is the predominant GATA family member expressed in primary prechondrocyte cultures, and is specifically expressed in precartilaginous condensations in both the axial and appendicular skeleton of mouse embryos [26]. In order to determine the expression pattern of all GATA transcription factors (i.e., GATA1-6) and their transcriptional co-factors (i.e., Friends of GATA (FOG1/2) [27]) in the early mouse limb bud, we dissected the anterior and posterior regions of limb buds isolated from E11.5 mouse embryos, and assayed expression of both GATA and FOG family members by RT-qPCR. This analysis revealed that both GATA4 and GATA6 are preferentially expressed in the anterior regions of the limb buds. While GATA4 expression is highly enriched in the anterior region of the forelimb bud (Figure 1A, lanes 13–16), GATA6 is expressed approximately 2.5 fold greater in the anterior region of both forelimbs and hindlimbs of E11.5 mouse embryos (Figure 1A, lanes 21–24). In contrast, other GATA family members and FOG1/2 co-factors are expressed at approximately equivalent levels in both the anterior and posterior regions of the developing limb buds (Figure 1A). To confirm these findings, we performed whole mount in situ hybridization (WISH) analysis employing probes for either the 3′UTR [28] or exon 2 [29] of mouse GATA6. WISH analysis with either probe indicated that GATA6 is first expressed in the distal mesenchyme of the forelimb starting at E10.5 (Figure 1Ba and Figure S1a), and is expressed in the anterior mesenchyme of both the forelimb and hindlimb buds at E11.5 (Figures 1Bb and 1Be; Figures S1b and S1e). By E12.5, GATA6 expression ceases in the anterior mesenchyme in both fore- and hindlimb buds (Figures 1Bc and 1Bf; Figures S1c and S1f). To confirm that the observed in situ hybridization signal for GATA6 was specific, we performed WISH for GATA6 expression (employing an exon 2 probe) in mouse embryos in which exon 2 of *GATA6* was conditionally deleted from their limb buds [30]. In the analyzed mutant embryos, the in situ hybridization signal for exon 2 of GATA6 was specifically lost in the anterior mesenchyme of the limb buds (Figure S1a'–f'), indicating that the WISH signal for GATA6 in the anterior mesenchyme of the limb buds is specifically detecting endogenous GATA6 expression.

Using two different sets of primers to amplify either GATA4, GATA5 or GATA6 cDNAs in RT-qPCR analyses, we observed that GATA6 transcripts in the anterior hindlimb of E11.5 wild type embryos, were consistently detected at a C_t_ value (which is inversely proportional to mRNA abundance) that was 2 cycles lower than the C_t_ value for GATA5 transcripts, and at 5 cycles lower than the C_t_ value for GATA4 transcripts; thus suggesting that GATA6 is expressed in the hindlimb bud at significantly higher levels than these other GATA factors. Additionally, we compared the relative expression of either GATA1, GATA2, or GATA3 in limb buds to that of GATA6 by RT-qPCR analysis, and found that GATA6 was detected at a C_t_ value that was at least 2 cycles lower than the C_t_ values for GATAs 1–3. These findings, taken together with our observation that GATA6 is preferentially expressed in the anterior mesenchyme of the hindlimb bud, prompted us to focus our attention on the role of GATA6 in hindlimb development. *GATA6* knock-out mice display early embryonic lethality at E6.5, due to a failure in endoderm differentiation [31], [32]. Therefore, to evaluate the effect of *GATA6* loss on limb development, we performed conditional deletion of the *GATA6* gene in mouse limb bud mesenchyme using Cre recombinase driven by the Prx1 regulatory regions (i.e., *Prx1-Cre*). *Prx1-Cre* efficiently drives Cre-mediated recombination in the developing limb buds and can induce recombination at approximately E9.5 in the forelimb and E10.5 in the hindlimb [33]. We created mice homozygous for a conditional allele of *GATA6* (*GATA6^fl/fl^*) [30] and heterozygous for *Prx1-Cre*. In contrast to WT mice, the in situ hybridization signal for exon 2 of GATA6 was specifically lost in the anterior mesenchyme of the limb buds in *Prx1-Cre; GATA6^fl/fl^* mice (Figure S1a'–f'), in which exon 2 of both *GATA6* alleles is flanked by loxP binding sites [30]. Interestingly, we observed that *Prx1-Cre; GATA6^fl/fl^* animals displayed hindlimb preaxial polydactyly (Figure 1C, right panel, compare with the control left panel), with a variable number of supernumerary digits ranging from 1 extra digit to 2 extra digits with additional phalanges. Consistent with the efficient deletion of the *GATA6^fl/fl^* alleles in the limb buds of *Prx1-Cre; GATA6^fl/fl^* mice (Figure S1a'–f'), we noted that the penetrance of hindlimb polydactyly was quite robust, with 100% of these animals displaying this phenotype (n = 20). In contrast to the hindlimbs, the forelimbs of *Prx1-Cre; GATA6^fl/fl^* mice did not display an abnormal number of digits (Figure S2A), suggesting that other factors (i.e., GATA4) may play a redundant role with GATA6 to block polydactyly specifically in the forelimb (discussed below).

### Loss of *GATA6* in the hindlimb buds induces ectopic expression of hedgehog responsive genes

**Figure 2:**
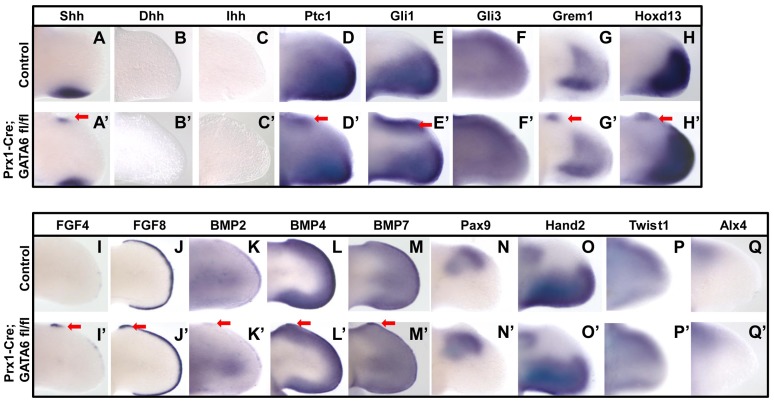
Loss of GATA6 in the hindlimb bud induces ectopic expression of hedgehog responsive genes. Whole mount in situ hybridization analysis of gene expression in E11.5 hindlimb buds from either control (A–Q) or *Prx1*-Cre; *GATA6* ^fl/fl^ (A'–Q') embryos. Ectopic gene expression is indicated by red arrows. At least 4 *Prx1*-Cre; *GATA6* ^fl/fl^ limbs were analyzed for each in situ probe and representative limbs are shown.

Because ectopic activation of the hedgehog signaling pathway in the anterior mesenchyme of the limb bud has been documented to induce polydactyly [1], [12], [13], [14], [34], [35], [36], our finding that loss of *GATA6* in the limb buds induced polydactyly in the hindlimbs raised the prospect that GATA6 functions in the anterior region of the hindlimb bud mesenchyme to repress the hedgehog signaling pathway. To investigate this possibility, we performed WISH analysis to assay the expression of transcriptional targets of hedgehog signaling in the limb bud. We observed that E11.5 *Prx1-Cre; GATA6^fl/fl^* embryos displayed detectable ectopic expression of both Shh (in 3/4 hindlimbs) and its transcriptional targets Ptc1 (in 4/4 hindlimbs) and Gli1 (in 4/4 hindlimbs) in the anterior mesenchyme of their hindlimb buds (Figure 2A', 2D', 2E', red arrows). Since ectopic expression of hedgehog family members other than Shh could potentially cause a polydactylous phenotype [37], we investigated if either ectopic Desert Hedgehog (Dhh) or Indian Hedgehog (Ihh) was expressed in *GATA6* mutant limbs. WISH analysis of E11.5 *Prx1-Cre; GATA6^fl/fl^* embryos did not detect ectopic expression of either Ihh or Dhh in the developing hindlimb buds of these animals (in 4/4 hindlimbs for each probe) (Figure 2B'and 2C'). Grem1 and Hoxd13, whose spatial expression in the limb bud is regulated (directly or indirectly) by Gli3R (reviewed in [4], [5]), were also ectopically expressed in the anterior mesenchyme in hindlimb buds of *GATA6* mutants (in 4/4 hindlimbs for each probe) (Figure 2G' and 2H', red arrows). In contrast, the forelimbs of these animals (which develop normally; Figure S2A) failed to display ectopic expression of these genes outside of their usual expression domain, which normally is restricted to the posterior limb bud mesenchyme (Figure S2B).

In mice that display polydactyly, such as in either *Gli3^−/−^, Gli3^P1-4/P1-4^, or Prx1-Cre; Ptc1^fl/fl^* animals, ectopic expression of both Shh and Grem1 in the anterior region of the limb bud results in the anterior expansion of FGF4 expression in the AER [12], [13], [14], [38]. Therefore, we examined if *Prx1-Cre; GATA6^fl/fl^* mice similarly displayed an expansion in the expression of both FGF4 and FGF8 in the anterior AER. Indeed, WISH analysis with either FGF4 or FGF8 probes indicated an ectopic anterior up-regulation of FGF4 expression and an anterior expansion of FGF8 expression in the AER of *GATA6* mutant E11.5 hindlimbs (in 4/4 hindlimbs for each probe) (Figure 2I' and 2J', red arrows). The expression domains of BMP2, BMP4, and BMP7 in the AER were also expanded anteriorly in the hindlimbs of *Prx1-Cre; GATA6^fl/fl^* mice (in 4/4 hindlimbs for each probe) (Figure 2K', 2L', 2M', red arrows). In contrast, neither expression of Hand2, a posterior mesenchymal marker, which is known to regulate Shh expression [23], [24], nor expression of either Gli3, Twist1 or Alx4, which are normally expressed in the anterior region of the limb bud mesenchyme and mutations in which cause preaxial polydactyly [12], [13], [19], [20], [21], was altered in the hindlimbs of *Prx1-Cre; GATA6^fl/fl^* mice (in 4/4 hindlimbs for each probe) (Figure 2O', 2F', 2P' and 2Q'). In mice with conditional knock-out of *GATA6* in their limbs, the expression of the anterior mesenchymal marker Pax9 was excluded from the region of the hindlimb bud which displayed ectopic expression of SHH target genes (in 4/4 hindlimbs) (Figure 2N'), and correlates with the exclusion of Pax9 expression from the mesenchyme of the forming supernumerary digits in the anterior region of these mutant limb buds. These results indicate that loss of *GATA6* in the hindlimb buds induced the ectopic expression of both Shh and transcriptional targets of hedgehog signaling in the anterior mesenchyme of the hindlimb buds, which consequently resulted in the expression of FGF family members in the anterior AER of these structures.

### Forced-expression of GATA6 throughout the limb bud represses induction and/or maintenance of Shh, Gli1 and Grem1 expression

Because loss of GATA6 from the hindlimb bud induced ectopic expression of Shh and its transcriptional targets, resulting in preaxial polydactyly, we evaluated whether forced expression of GATA6 throughout the limb bud would conversely repress expression of Shh and its targets. To drive exogenous GATA6 expression specifically in limb bud mesenchymal cells we generated transgenic mice containing a mouse GATA6 cDNA positioned downstream of reiterated Tet binding sites (*GATA6-Tg*). This transgenic animal was in turn mated to an animal containing both a reverse tetracycline transactivator (rtTA; which binds to Tet binding sites only in the presence of doxycycline), positioned downstream of a “floxed” STOP transcription cassette (*ROSA26-rtTA* [39]), plus Cre recombinase driven by the *Prx1* regulatory sequences (*Prx1-Cre*). *Prx1-Cre* drives recombination and thus expression of the reverse tetracycline transactivator (rtTA) in limb bud mesenchymal cells. Doxycyline was administered in the drinking water to the dams that carried these triple transgenic animals (i.e., *Prx1-Cre; ROSA26-rtTA; GATA6-Tg*) beginning at E4.5, to induce the transcriptional activity of the reverse tetracycline transactivator (rtTA), which in turn induced the expression of ectopic mouse GATA6 in the limb bud mesenchyme.

**Figure 3:**
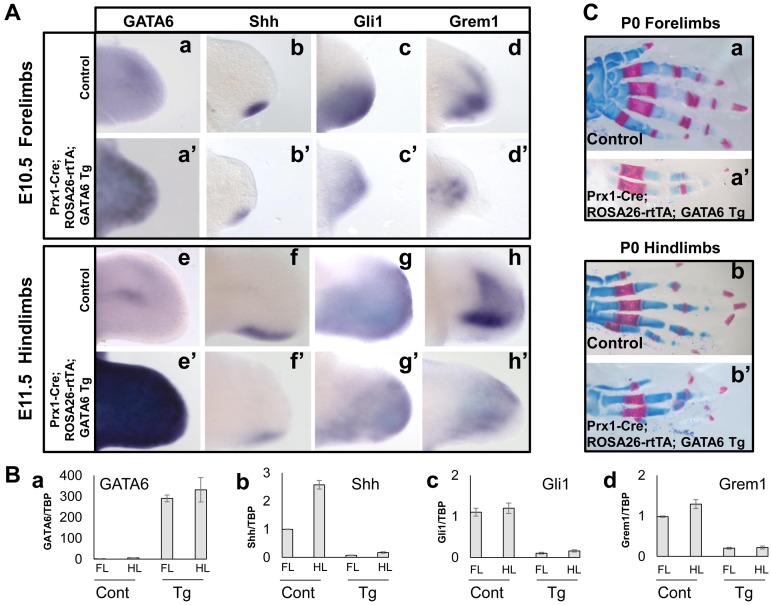
Transient forced expression of GATA6 throughout the developing limb bud represses induction of Shh, Gli1, and Grem1, and results in loss of digits. (**A**) Whole mount in situ hybridization analysis of gene expression in E10.5 forelimb and E11.5 hindlimb buds from either control (a–d, e–h) or *Prx1-Cre; ROSA26-rtTA; GATA6-Tg* (a'–d', e'–h') transgenic mouse embryos (that had been administered doxycycline beginning at E4.5); at least 6 limbs were analyzed for each probe, representative samples are shown. (**B**) RT-qPCR analysis of gene expression in E11.5 fore- or hindlimb buds isolated from either a transgenic (Tg) *Prx1*-Cre; *ROSA26*-rtTA; *GATA6*-*Tg* embryo (that had been administered doxycycline beginning at E4.5) or a littermate control (Cont) embryo. The value (i.e., transcript examined relative to GAPDH) of the 1st sample in each graph (FL control) was set to 1.0; and the other samples normalized to that value for each of the graphs. (**C**) Alcian Blue/Alizarin Red staining of either P0 control (a, b) or *Prx1-Cre; ROSA26-rtTA; GATA6-Tg* (a', b') mouse forelimbs (a, a') and hindlimbs (b, b') (that had been exposed to doxycycline from E4.5 until E11.5). At least 10 P0 *Prx1-Cre; ROSA26-rtTA; GATA6-Tg* animals were analyzed and digit loss was observed with 100% penetrance in both forelimbs and hindlimbs in these mutants; milder mutants are shown.

The triple transgenic embryos and controls were harvested at either E10.5 or E11.5 and exogenous GATA6 expression was confirmed by whole mount in situ hybridization analysis in both forelimbs (Figure 3A, compare panel a' with a) and hindlimbs (Figure 3A, compare panel e' with e). Interestingly, ectopic expression of GATA6 decreased the expression of Shh, Gli1, and Grem1 in both the forelimbs and hindlimbs of *Prx1-Cre; ROSA26-rtTA; GATA6-Tg* animals (in 6/6 limb buds analyzed for each marker) (Figure 3A, compare panels b'–d' to b–d, and f'–h' to f–h, respectively). Consistent with the necessity for Shh to promote expression of FGFs in the AER and consequent limb bud outgrowth [3], the limbs buds of the triple transgenic embryos were diminished in size (Figure 3A). To better quantitate the effects of ectopic GATA6 on gene expression in the limb bud, limb buds were harvested from either an E11.5 *Prx1-Cre; ROSA26-rtTA; GATA6-Tg* animal (that had been administered doxycycline beginning at E4.5) or a control littermate, and gene expression was assayed by RT-qPCR. We observed that expression of Shh, Gli1 and Grem1 was drastically reduced in both the forelimbs and hindlimbs of the *Prx1-Cre; ROSA26-rtTA; GATA6-Tg* animal in comparison to the limb buds of the control littermate (Figure 3B).

Lastly, we determined whether formation of the skeletal elements was affected in triple transgenic mice transiently exposed to doxycycline. Triple transgenic animals (and their littermate controls) were exposed to doxycycline from E4.5 until E11.5 to allow a transient pulse of GATA6 in their developing limb buds. Alcian Blue/Alizarin Red staining of the skeletal tissue of such animals sacrificed at P0 indicated that a transient pulse of GATA6 in the limb buds drastically affected the subsequent formation of the autopod, with a striking reduction in the number of digits in both the forelimbs (Figure 3C, compare a' to a) and the hindlimbs (Figure 3C, compare b' to b). In all P0 *Prx1-Cre; ROSA26-rtTA; GATA6-Tg* animals that were analyzed (n = 10), digit loss was 100% penetrant in both forelimbs and hindlimbs in these mutants. These morphological effects are consistent with a transient repression of Shh and its downstream targets in the developing limb buds of these animals at earlier stages of development (Figure 3A).

### Gli3 processing is not significantly altered throughout the anterior region of *Prx1-Cre; GATA6^fl/fl^* hindlimb buds

**Figure 4:**
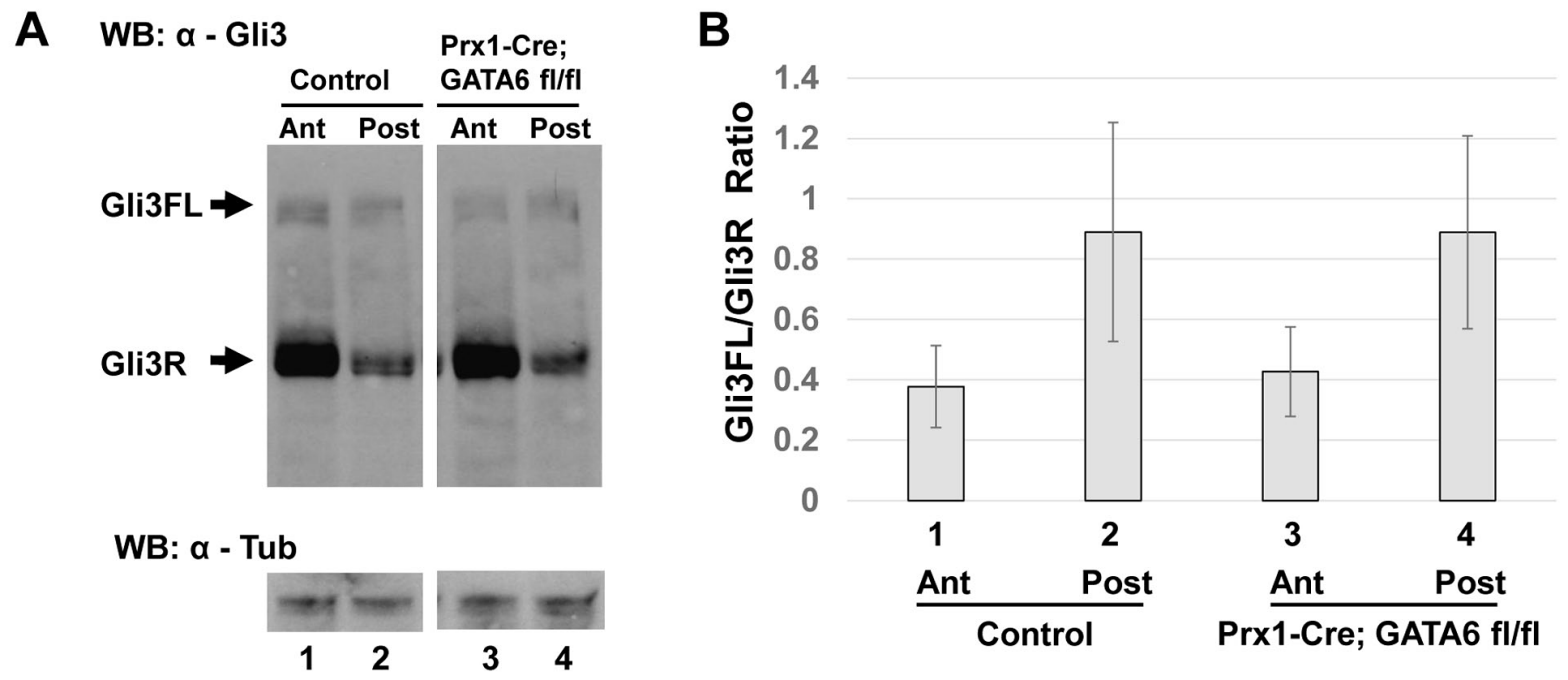
Conditional deletion of *GATA6* does not significantly alter Gli3 processing in the hindlimb bud. (**A**) Western blot analysis of Gli3FL and Gli3R steady state levels in anterior (lanes 1 and 3) or posterior (lanes 2 and 4) halves of E11.5 hindlimb buds isolated from either control (lanes 1 and 2) or *Prx1*-Cre; *GATA6* ^fl/fl^ (lanes 3 and 4) mouse embryos. α–Tubulin is used as a loading control. (**B**) Quantification of Gli3FL/Gli3R ratios (by densitometry of Western Blots) in anterior or posterior halves of E11.5 hindlimb buds isolated from either littermate controls (lanes 1 and 2) or *Prx1*-Cre; *GATA6* ^fl/fl^ (lanes 3 and 4) animals, 12 embryos of each genotype were analyzed. Error bar indicates standard deviation, n = 12.

Shh signaling in the limb bud is transduced intracellularly by a signaling cascade that inhibits processing of full length Gli3 (Gli3FL) activator into its repressor form (Gli3R). In wild-type limbs, the ratio of Gli3FL to Gli3R decreases from posterior (where *Shh* is expressed) to anterior regions of the limb bud, and is crucial for correct limb patterning (reviewed in [40]). Because loss of GATA6 in the hindlimb bud resulted in up-regulation of Gli3 target genes (such as Gli1) in the anterior hindlimb bud mesenchyme, we wondered whether the ratio of Gli3FL to Gli3R was altered in the hindlimb buds of *Prx1-Cre; GATA6^fl/fl^* mice. To examine this possibility, hindlimb buds were isolated from either E11.5 *Prx1-Cre; GATA6^fl/fl^* embryos (which display hindlimb polydactyly) or their littermate *GATA6^fl/+^* controls (which display a normal number of digits in all their limbs), cut into anterior and posterior halves, and protein extracts made from these tissues were separated by SDS-PAGE, Western blotted, and probed with an antisera against the N-terminus of Gli3 that recognizes both the 190 kDa Gli3FL protein as well as the 83–86 kDa Gli3R processed form of this protein. Importantly, expression of proteins recognized by this antibody were decreased by RNAi-mediated knock-down of Gli3 in NIH3T3 cells (Figure S3), attesting to the identity of these immuno-reactive proteins as bona fide Gli3 isoforms. Consistent with prior studies [41], [42], we found that Gli3R specifically accumulated in the anterior half of wild-type E11.5 hindlimb buds (Figure 4A, compare lanes 1 and 2). Surprisingly, we found that the relative steady state ratio of Gli3FL: Gli3R in either the anterior or posterior regions of hindlimb buds isolated from E11.5 *Prx1-Cre; GATA6^fl/fl^* embryos was not significantly different from their littermate controls (Figure 4A, compare lane 3 with lane 1, and lane 4 with lane 2; the results of similar Western analyses of the hindlimbs of 12 embryos of each genotype are quantitated in Figure 4B). Loss of GATA6 induced ectopic expression of Shh in the anterior region of the hindlimbs of E11.5 *Prx1-Cre; GATA6^fl/fl^* embryos, which presumably promoted the accumulation of Gli3FL at the expense of Gli3R in adjacent cells. Interestingly however, our Western analyses indicated that loss of GATA6 in the hindlimb buds of *Prx1-Cre; GATA6^fl/fl^* embryos did not significantly alter the steady state ratio of Gli3FL:Gli3R to a sufficient degree in the anterior hindlimb bud to be picked up by this technique. Thus, these findings suggest that the level of ectopic Shh induced by loss of GATA6 in the hindlimb bud may only alter the Gli3FL:Gli3R ratio in a relatively small number of cells that lie adjacent to the source of ectopic Shh.

### GATA6 binds to regulatory regions that control the expression of the *Gli1* gene

**Figure 5:**
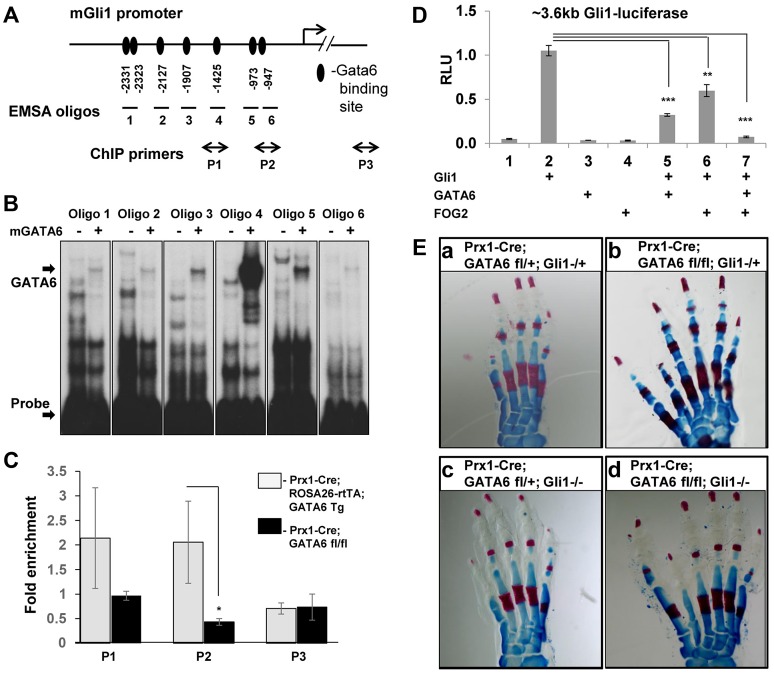
GATA6 binds to regulatory regions that control the expression of the mouse *Gli1* gene and blocks expression of reporters driven by this sequence. Loss of *Gli1* cannot rescue polydactyly induced by conditional deletion of *GATA6* in mouse hindlimbs. (**A**) GATA6 binding sites identified by EMSA analysis in the promoter of the mouse *Gli1* gene. Positions of GATA6 binding sites are indicated relative to the transcription start site of *Gli1*. Oligos 1–6 used to perform electrophoretic mobility shift essay (EMSA) are indicated. Positions of qPCR primers used in ChIP experiments are shown (P1–P3), P3 is a control pair of primers located ∼6 kb downstream of Gli1 start site. (**B**) EMSA employing in vitro translated mouse GATA6 with oligos from the *Gli1* promoter (displayed in A). Arrows point to the position of either the GATA6-oligo complexes or to the unbound labeled probes. (**C**) Chromatin IP to assay GATA6 interaction with the Gli1 promoter in limb buds isolated from either E11.5 *Prx1-Cre; ROSA26-rtTA; GATA6-Tg* animals, that had been pulsed with doxycycline from E4.5 until harvest, versus limb buds isolated from E11.5 *Prx1-Cre; GATA6^fl/fl^* mice. Positions of ChIP primers on the *Gli1* promoter are displayed in A. Chromatin was sheared to between 500–1000 bp prior to ChIP with anti-GATA6 antibody. *p<0.05; Student's test. Error bar indicates standard deviation, n = 3. (**D**) GATA6 blocks Gli1-dependent activation of Gli1-luciferase. NIH3T3 cells were co-transfected with a ∼3.6 kb Gli1-firefly luciferase reporter plus expression vehicles encoding either Gli, GATA6, or FOG2, as indicated. Firefly luciferase units were normalized to the expression of co-transfected SV40-renilla luciferase to obtain Relative Luciferase Units (RLU). *p<0.05, **p<0.01, ***p<0.001; Student's test. Error bar indicates standard deviation, n = 3. (**E**) Alizarin Red/Alcian blue staining of the hindlimbs of P0 mice of the indicated genotypes. At least 5 animals of each genotype were analyzed; representative samples are shown.

Because expression of Gli1 was significantly induced in the anterior region of the hindlimb buds in *Prx1-Cre; GATA6^fl/fl^* animals (Figure 2E') and relatively decreased in limb buds engineered to over-express GATA6 (Figure 3A and B), we examined whether GATA6 may directly bind to the promoter of the *Gli1* gene [43] and thereby repress its transcription. To test whether in vitro translated mouse GATA6 can bind to putative GATA6 binding sites [44] in the *Gli1* promoter, we performed electrophoretic mobility shift assays (EMSA). EMSA analysis of oligos containing putative GATA6 binding sites in a ∼3.6 kb sequence encompassing the *Gli1* promoter [43], identified seven GATA6 binding sites upstream of the *Gli1* transcriptional start site (Figure 5A and 5B). Mutation of these GATA6 binding sites (changing GATA/GATT to GGTA/GGTT) completely eliminated GATA6 binding to these oligos (Figure S4A and S4B), confirming the specificity of GATA6 interaction with these sequences of the *Gli1* promoter. Interestingly, the highest affinity GATA6 binding site we identified in the *Gli1* promoter (in oligo 4; Figure 5A and 5B) is conserved in the homologous human sequence (Figure S4C).

To examine whether GATA6 associates with the *Gli1* regulatory region *in vivo*, we performed chromatin IP for GATA6 with limb buds isolated from E11.5 *Prx1-Cre; ROSA26-rtTA; GATA6-Tg* animals that had been pulsed with doxycycline from E4.5 until harvest. As a negative control, we employed limb buds isolated from E11.5 *Prx1-Cre; GATA6^fl/fl^* mice (which lack expression of GATA6 in their limb buds). We found that GATA6 was enriched on chromatin encompassing the GATA binding sites upstream of the *Gli1* gene in limb buds specifically isolated from E11.5 *Prx1-Cre; ROSA26-rtTA; GATA6-Tg* animals that had been programmed to express exogenous GATA6 (Figure 5A and 5C).

Next, we evaluated whether GATA6 could directly modulate the expression of a luciferase reporter (∼3.6 kb Gli1-luciferase) driven by 3.6 kb of regulatory sequences upstream of the mouse *Gli1* gene. Consistent with the ability of this sequence to respond to hedgehog signals [43], expression of ∼3.6 kb Gli1-luciferase was markedly enhanced by co-transfection of NIH3T3 cells with an expression vehicle encoding Gli1 (Figure 5D, lane 2). Interestingly, co-transfection of GATA6 significantly down-regulated induction of this reporter by Gli1 (Figure 5D, lane 5), suggesting that GATA6 can directly inhibit *Gli1* promoter activity. GATA factors can either be transcriptional activators or repressors; their repressor activity requiring interaction with either of the transcriptional co-factors FOG1 or FOG2 (reviewed in [27], [45]). We found that co-transfection of GATA6 plus FOG2 could synergistically repress induction of ∼3.6 kb Gli1-luciferase by co-transfected Gli1 in NIH3T3 cells (Figure 5D, lanes 5–7), suggesting that GATA6 works together with endogenous FOG co-factors to repress ectopic expression of Gli1 in the anterior region of the hindlimb buds.

### Loss of Gli1 does not block polydactyly in mice lacking GATA6 in their hindlimbs

Knock-in of *Gli1* into the *Gli2* locus has been demonstrated to enhance the severity of polydactyly in *Gli3* ^+/−^ mice [46], suggesting that ectopic expression of Gli1 in the anterior mesenchyme of the hindlimb buds in *Prx1-Cre; GATA6^fl/fl^* mice may contribute to this phenotype. In addition, because GATA6 can both bind to the *Gli1* promoter *in vivo* and inhibit activation of a luciferase reporter driven by this regulatory sequence *in vitro*, we wondered if the hindlimb polydactyly observed in *Prx1-Cre; GATA6^fl/fl^* animals may be a consequence of derepressed expression of Gli1 in the anterior mesenchyme of the hindlimbs in these animals. To examine this possibility, we generated mice lacking both *GATA6* and *Gli1* in the mesenchyme of their limb buds. As expected, control heterozygous animals (*Prx1-Cre; GATA6^fl/+^; Gli1^−/+^*) or animals lacking only Gli1 (*Prx1-Cre; GATA6^fl/+^; Gli1^−/−^*) had normal limbs (Figure 5Ea and 5Ec). In contrast, animals homozygous for both *GATA6* and *Gli1* loss in their limb buds (*Prx1-Cre; GATA6^fl/fl^;Gli1^−/−^*) displayed hindlimb polydactyly, similar to hindlimbs lacking only *GATA6* (*Prx1-Cre; GATA6^fl/fl^;Gli1^+/−^*) (Figure 5Ed compare with 5Eb). Thus, loss of *Gli1* failed to reverse the hindlimb polydactyly in *GATA6* conditional knockout animals, indicating that ectopic expression of Gli1 in the anterior mesenchyme of hindlimbs lacking *GATA6* is apparently not necessary for the formation of extra digits.

### GATA6 binds to regulatory regions that control the expression of the *Shh* gene

**Figure 6:**
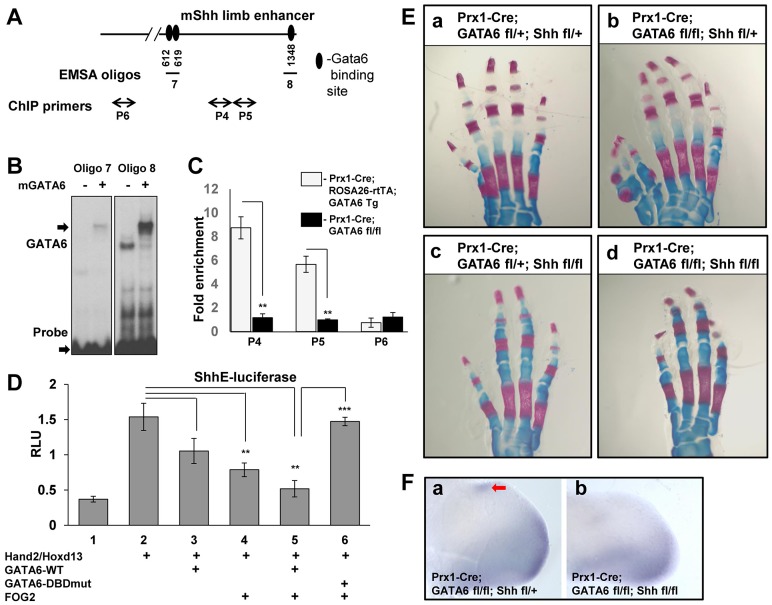
GATA6 binds to regulatory regions that control the expression of the mouse *Shh* gene and blocks expression of reporters driven by this sequence. Conditional deletion of *Shh* from the mouse limb bud rescues polydactyly induced by conditional loss of *GATA6* in the mouse hindlimbs. (**A**) GATA6 binding sites identified by EMSA in the conserved region (ShhE) of the mouse *Shh* limb bud enhancer ZRS. Position of GATA6 binding sites are indicated relative to the first HindIII site in the ZRS sequence [16]. Oligos 7 and 8 used to perform EMSA are indicated. Positions of qPCR primers used in CHIP experiments are shown (P4–P6), P6 is a control pair of primers located ∼6 kb upstream of the ShhE sequence. (**B**) Electrophoretic mobility shift assays of in vitro translated GATA6 with oligos from the ShhE region of the ZRS enhancer. Arrows point to the position of either the GATA6-oligo complexes or to the unbound labeled probes. (**C**) Chromatin IP to assay GATA6 interaction with the conserved region (ShhE) of the mouse *Shh* limb bud enhancer (ZRS) in limb buds isolated from either E11.5 *Prx1-Cre; ROSA26-rtTA; GATA6-Tg* animals, that had been pulsed with doxycycline from E4.5 until harvest, versus limb buds isolated from E11.5 *Prx1-Cre; GATA6^fl/fl^* mice. Positions of primers on the ShhE enhancer are displayed in A. Chromatin was sheared to between 500–1000 bp prior to ChIP with anti-GATA6 antibody. **p<0.01; Student's test. Error bar indicates standard deviation, n = 3. (**D**) GATA6 and FOG2 cooperatively block Hand2/Hoxd13-mediated induction of a luciferase reporter driven by the conserved region of the ZRS enhancer (ShhE) that drives *Shh* expression in the limb bud. NIH3T3 cells were co-transfected with an ShhE-firefly luciferase reporter plus expression vehicles encoding either Hand2, Hoxd13, GATA6-WT, GATA6-DNA binding domain mutant (GATA6DBDmut), or FOG2, as indicated. Firefly luciferase units were normalized to the expression of co-transfected SV40-renilla luciferase to obtain Relative Luciferase Units (RLU). *p<0.05, **p<0.01, ***p<0.001; Student's test. Error bar indicates standard deviation, n = 3. (**E**) Alizarin Red/Alcian blue staining of the hindlimbs of P0 mice of the indicated genotypes. At least 5 animals of each genotype were analyzed; representative samples are shown. (**F**) Whole mount in situ hybridization analysis of Gli1 expression in E11.5 *Prx1-Cre; GATA6^fl/fl^; Shh^fl/fl^* embryos vs. E11.5 *Prx1-Cre; GATA6^fl/fl^; Shh^fl/+^* embryos. Four limbs of each genotype were analyzed; representative images are shown. Ectopic Gli1 expression is noted by the red arrow.

The above findings suggest that GATA6 normally blocks the formation of hindlimb polydactyly by repressing expression of other genes in the hindlimb bud in addition to Gli1. One plausible candidate that GATA6 might directly repress is *Shh*, as we observed ectopic expression of this gene in the anterior mesenchyme of hindlimbs deleted for GATA6 (Figure 2A'), and over-expression of exogenous GATA6 in limb buds repressed expression of Shh (Figures 3A and 3B). To explore this possibility, we examined whether GATA6 interacts with the regulatory element that drives *Shh* expression in the limb buds. The *Shh* limb bud-specific enhancer sequence, termed ZRS, lies 1 Mb upstream of the *Shh* coding region and can drive ZPA-specific LacZ expression in transgenic mice [16]. Within the ZRS lies a very conserved region of ∼0.8 kb, termed ShhE [16], [47]. Therefore, we examined whether mouse GATA6 can bind to the ShhE regulatory sequence by EMSA analysis, with in vitro translated GATA6 protein and oligos containing putative GATA6 binding sites identified in the ShhE. We discovered three GATA6 binding sites in this portion of the ZRS enhancer (Figure 6A and 6B). Mutation of these GATA6 binding sites (from GATA/GATT to GGTA/GGTT) eliminated GATA6 binding to these oligos (Figure S4D and S4E), confirming sequence specificity of GATA6 binding to these sites. Interestingly, two of the three GATA6 binding sites we identified in the murine ShhE sequence are conserved in the homologous human sequence (Figure S4F). Seeing that GATA6 can bind to conserved GATA binding sites in the ShhE sequence *in vitro*, we evaluated whether GATA6 could bind to these sequences *in vivo* by performing chromatin IP for GATA6 with limb buds isolated from E11.5 *Prx1-Cre; ROSA26-rtTA; GATA6-Tg* animals that had been pulsed with doxycycline from E4.5 until harvest. As a negative control, we employed limb buds isolated from E11.5 *Prx1-Cre; GATA6^fl/fl^* mice (which lacked GATA6 in their limb buds). We found that GATA6 is indeed associated with chromatin encompassing the ShhE sequence specifically in limb buds isolated from *Prx1-Cre; ROSA26-rtTA; GATA6-Tg* animals that had been programmed to express exogenous GATA6 (Figures 6A and 6C)

Finally, we examined whether GATA6 could modulate the expression of a luciferase reporter driven by the ShhE sequence (ShhE-luciferase [47]), which has been demonstrated to drive Shh expression in the limb bud mesenchyme in response to the activities of Hox proteins [47] and Hand2 [48]. Consistent with the notion that Shh is positively regulated by both Hox factors [47], [49] and Hand2 [48] in the developing limb bud, co-transfection of expression vehicles encoding Hoxd13 and Hand2 significantly boosted expression of ShhE-luciferase in co-transfected NIH3T3 cells (Figure 6D, lane 2). As we had observed with Gli1-luciferase, co-transfection of GATA6 plus FOG2 synergistically repressed induction of the ShhE-luciferase reporter in NIH3T3 cells (Figure 6D, lanes 3–5). To examine whether GATA6:DNA interaction is necessary for repression of ShhE-luciferase by co-transfected GATA6, we evaluated whether a mutant form of GATA6 that cannot directly bind to DNA (GATA6-DBDmut) could repress the expression of ShhE-luciferase. We found that in contrast to wild-type GATA6, co-transfection of GATA6-DBDmut (together with FOG2) failed to attenuate the induction of ShhE-luciferase by co-transfected Hoxd13 and Hand2 (Figure 6D, lane 6), and indeed blocked the ability of exogenous FOG2 to repress expression of this reporter (compare Figure 6D, lanes 4 and 6). These results are consistent with the notion that GATA6-DBDmut, which does not directly bind to DNA, can nonetheless interact with FOG2 (off the DNA), and thereby sequester this co-factor from interaction with endogenous GATA factors that can directly bind to the ShhE sequence. In summary, these findings suggest that GATA6 represses ectopic expression of Shh in the anterior region of the hindlimb buds by directly binding to the GATA binding sites in the ShhE and thereby recruiting FOG co-factors to this regulatory sequence.

### Loss of Shh blocks polydactyly in mice lacking GATA6 in their hindlimbs

*Gli3* ^−/−^ mice, *Gli3^P1-4/P1-4^* mice, and *Alx4* ^−/−^ mice all exhibit polydactyly, and all display ectopic expression of Shh in the anterior region of the developing limb bud [12], [13], [14], [50]. Interestingly however, polydactyly is still induced in *Gli3* ^−/−^ or *Gli3^P1-4/P1-4^* mice that have been engineered to also lack Shh expression (i.e., *Gli3* ^−/−^; *Shh* ^−/−^ mice and *Gli3^P1-4/P1-4^; Shh^−/−^* mice) [12], [13], [14], indicating that ectopic Shh expression in the anterior limb bud mesenchyme observed in these mice is not necessary to effect polydactyly. In contrast, *Alx4* ^−/−^; *Shh* ^−/−^ mice display a phenotype that resembles *Shh*-deficiency alone, with only a single digit in each limb [13]. Thus, polydactyly can be induced either in a Shh-dependent fashion such as occurs in *Alx4* ^−/−^ mice, or in a Shh-independent fashion following loss of Gli3R function, such as occurs in *Gli3* ^−/−^ mice and *Gli3^P1-4/P1-4^* mice. To examine whether Shh is necessary to induce polydactyly in hindlimbs lacking *GATA6* we generated *Prx1-Cre; GATA6^fl/fl^; Shh^fl/fl^* mice, in which both *GATA6* and *Shh* were conditionally knocked out from the limb bud mesenchyme. Consistent with prior work indicating a crucial role for Shh in both limb bud A-P patterning and outgrowth [3], [12], conditional deletion of *Shh* in limb buds with *Prx1*-Cre produced mice with only 4 digits in their hindlimbs (*Prx1-Cre; GATA6^fl/+^; Shh^fl/fl^* mice; Figure 6Ec). While conditional deletion of *GATA*6 led to polydactyly in hindlimbs engineered to have one intact allele of Shh (*Prx1-Cre; GATA6^fl/fl^; Shh^fl/+^* mice; Figure 6E, compare a and b), deletion of *GATA6* failed to increase the number of digits in limbs engineered to conditionally delete both alleles of *Shh* (*Prx1-Cre; GATA6^fl/fl^; Shh^fl/fl^* mice; Figure 6E, compare c and d). Consistent with these effects on digit formation, E11.5 *Prx1-Cre; GATA6^fl/fl^; Shh^fl/fl^* embryos lack ectopic expression of Gli1 in the anterior mesenchyme of their hindlimb buds (Figure 6Fb), which is present in the hindlimb buds of E11.5 *Prx1-Cre; GATA6^fl/fl^; Shh^fl/+^* embryos (Figure 6Fa, red arrow). These findings indicate that deletion of *Shh* can block both the ectopic expression of hedgehog responsive genes in the anterior mesenchyme of hindlimb buds lacking *GATA6* and the subsequent occurrence of polydactyly in conditional *GATA6* knockout mice. Thus, ectopic expression of Shh is necessary for development of polydactyly in hindlimbs lacking *GATA6*.

## Discussion

### GATA6 plays a crucial role in repressing ectopic expression of Shh in the anterior mesenchyme of the hindlimb bud

**Figure 7:**
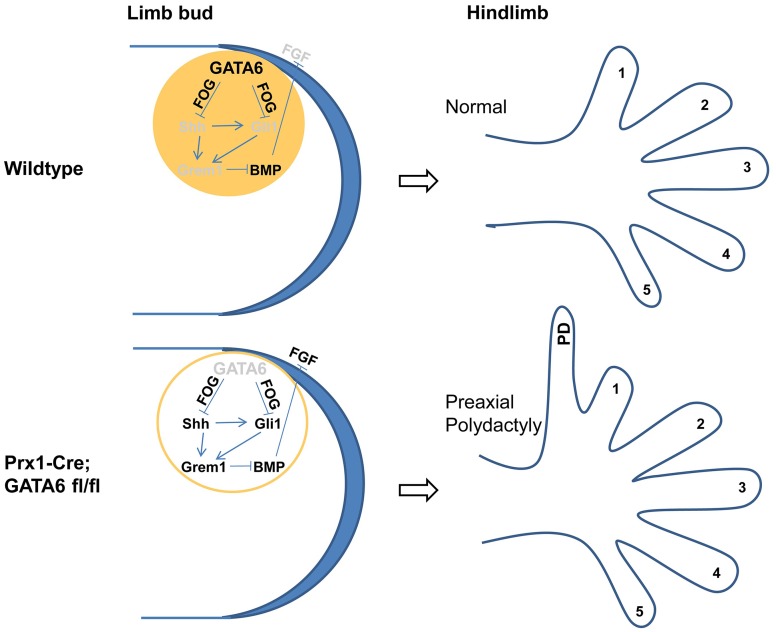
A model for GATA6 action in the anterior region of developing hindlimb buds. GATA6 is expressed in the anterior portion of the developing mouse hindlimb bud (displayed in top figures), and specifically represses polydactyly (PD) in the hindlimb by directly blocking anterior expression of Shh (most likely in a FOG-dependent fashion), and thus represses ectopic expression of both direct Shh transcriptional targets (Gli1 and Grem1) in anterior limb bud mesenchyme and indirect targets (FGF4/8) in the anterior AER. In hindlimb buds engineered to lack GATA6 (displayed in bottom figures), ectopic expression of Shh in the anterior mesenchyme induces expression of both Gli2/3 FL and Gli1 which activate expression of the BMP antagonist Grem1, which in turn derepresses expression of FGF4/8 in the anterior region of the apical ectodermal ridge. Thus, as a consequence of deletion of GATA6 in *Prx1-Cre; GATA6^fl/fl^* animals, the hindlimbs of these animals display preaxial polydactyly. Expressed genes are indicated by black font; knocked-out or repressed genes are indicated by grey font.

We have found that both GATA4 and GATA6 are preferentially expressed in the anterior region of the mesenchyme in developing limb buds. While both GATA factors are expressed in an A-P gradient in the forelimb buds (i.e., high anterior/low posterior), the hindlimb buds principally express GATA6 in an A-P gradient. Conditional loss of *GATA6* in limb buds results in ectopic expression of both Shh and Gli3 target genes, such as Gli1, Ptc1 and Grem1, specifically in the anterior mesenchyme of the hindlimb buds. In addition, loss of *GATA6* in the developing limbs results in the formation of preaxial polydactyly in the hindlimbs. In contrast, the combined loss of both *Shh* and *GATA6* in limb buds blocks both ectopic expression of Gli1 in the anterior mesenchyme of the hindlimb buds and the subsequent formation of hindlimb polydactyly, induced by loss of *GATA6* alone. These findings indicate that GATA6 plays a crucial role in repressing ectopic expression of Shh in the anterior mesenchyme of the hindlimb bud (shown schematically in Figure 7).

In mice, the only digit that does not require Shh signaling (digit 1) has two phalanges, while those that require Shh for their formation (digits 2–5) have three phalanges [12]. Interestingly, we observed that the dosage of Shh influenced the number of phalanges present on the extra digits in hindlimbs lacking GATA6; while ∼93% of the extra digits in the hindlimbs of P0 *Prx1-Cre; GATA6^fl/fl^; Shh^+/+^* mice displayed three phalanges (the remainder having two phalanges), only ∼70% of extra digits in the hindlimbs of P0 *Prx1-Cre; GATA6^fl/fl^; Shh^+/−^* mice displayed three phalanges. These results suggest that the dosage of ectopic Shh controls not only the formation but also the identity of the extra digits that form in hindlimbs lacking GATA6.

While loss of *GATA6* from the developing limbs induced ectopic expression of Shh and hedgehog transcriptional targets uniquely in the hindlimb, forced expression of GATA6 throughout the limb bud mesenchyme repressed expression of Shh and its targets in both forelimb and hindlimb buds. Because co-expressed GATA factors frequently display overlapping functions *in vivo* (reviewed in [51]), it seems plausible that both GATA4 and GATA6, which are both preferentially expressed in the anterior mesenchyme of the forelimb bud (Figure 1A), may redundantly block ectopic anterior expression of Shh in this structure.

### GATA6 works together with FOG co-factors to directly repress the expression of the Shh limb bud enhancer and the Gli1 promoter

How does GATA6 repress expression of Shh and hedgehog-inducible genes in the anterior mesenchyme of the limb bud? We have found that GATA6 is bound to chromatin encoding both the *Gli1* promoter (that responds to Shh signals) or to the *Shh* limb bud-specific enhancer (that drives limb bud-specific expression of the *Shh* gene) in the limb buds of transgenic mouse embryos expressing exogenous GATA6. Co-transfection of GATA6 repressed expression of luciferase reporters driven by either of these sequences. Interestingly, GATA6-mediated repression in NIH3T3 cells requires co-transfection with the transcriptional co-factor FOG2, which probably reflects the low levels of endogenous FOG expression in this cell line. Indeed endogenous FOG co-factors, which are known to bind to the transcriptional co-repressors NuRD and CtBP (reviewed in [45]) may similarly be rate limiting for exogenous GATA6 to repress expression of Shh, Grem1, and Gli1 in the limb bud, as we have found that these putative GATA6 targets were still expressed at low but detectable levels in limb buds programmed to express high amounts of exogenous GATA6 (Figures 3A and 3B). It appears that while GATA6 can both bind to a conserved Gli1 promoter-proximal sequence (as assayed by both EMSA and ChIP of limb buds) and repress expression of a luciferase reporter driven by this regulatory sequence, loss of GATA6 in the absence of Shh is not sufficient to derepress expression of Gli1 in the anterior region of the hindlimb bud. This finding is consistent with prior work indicating that Gli1 expression requires Gli2/3 FL activator function (see for instance [52], [53]), and suggests that Gli1 expression in the anterior domain of the hindlimb bud may be repressed by redundant mechanisms (i.e., Gli3R and GATA6 working in parallel). In addition, because Gli1 is not required to elicit a polydactylous phenotype in hindlimb buds lacking GATA6 (i.e., in *Prx1-Cre; GATA6^f/f^; Gli1^−/−^* mice), it argues against an important role for the Shh-dependent increase in Gli1 expression in causing this phenotype.

Interestingly, mutation of a putative GATA binding site (GGATA (second A to G mutation)) located in the Shh limb bud enhancer (i.e., ZRS homology region) has been identified in a family exhibiting autosomal dominant preaxial polydactyly (Belgian 2; [16]). However, in our hands, this putative GATA binding site did not display significant interaction with in vitro translated GATA6 by EMSA analysis (Figure S4G), which is consistent with in vitro binding assays indicating that GATA6 preferentially binds to (A/C/T)GATA versus GGATA [54]. While mutations in the GATA6 binding sites in either the human or mouse ZRS enhancer that induce polydactyly have not yet been identified, this does not exclude a role for GATA regulation of the ZRS enhancer. In addition, it is possible that GATA6 blocks ectopic expression of not only Shh itself, but also other Shh target genes in the limb bud (in addition to Gli1), in parallel with Gli3R. In this light, it will be interesting to determine whether GATA6 similarly binds to and represses activity of the regulatory regions that drive expression of Grem1 in the limb bud [55]. Lastly, our study does not rule out the possibility that GATA6 plays additional roles in limb bud patterning, independently of repressing expression of Shh and its downstream targets.

## Materials and Methods

### Plasmids and transgenic animal production

We used the following plasmids in this study: ∼3.6 kb Gli1-firefly luciferase (Dr. Ishii, RIKEN, Japan), pcDNA3-Hand2 (Rolf Zeller, University of Basel, Switzerland), pSG-Hoxd13 and pT81-mShhE (i.e., ShhE-firefly luciferase; Licia Selleri, Cornell University), pCS2-HA-mFOG-2 (Stuart Orkin, Harvard Medical School). Generation of pCS2-6MT-mGli1 is described in [56]. pcDNA3-GATA6-HA was generated by PCR-cloning of mouse GATA6 into pcDNA3.1+, using AflII and BamHI sites. An HA-tag was inserted at the C-terminus, in front of the GATA6 stop codon. A mutant form of GATA6 that was unable to directly bind to a GATA binding site (GATA6-DBDmut) was generated by mutating the 3^rd^ Cys of the C-terminal GATA6 zinc finger to Gly in pcDNA3-GATA6-HA. The GATA6 transgenic targeting construct was made by PCR-cloning the mouse GATA6 open reading frame into the pTRE-Tight plasmid (Clontech) using BamHI and ClaI restriction sites. Linearized transgenic constructs were injected into the pronuclei of fertilized oocytes to produce transgenic founders. The following primers were used to identify and genotype transgenic animals: CTCAAGTATTCAGGTCAAGACG and CTTTATTTGTAACCATTATAAGCTGC.

### Mice

All mice experiments were approved by the Institutional Animal Care and Use Committee (IACUC) of Harvard Medical School. *GATA6* ^fl^, *Prx1*-Cre, *Gli1* ^−^, and *Shh* ^fl^ alleles were described previously [30], [33], [57], [58]. Genotyping of both embryos and postnatal animals was performed by PCR analysis of tail biopsies. In order to generate *Prx1*-Cre; *GATA6* ^fl/fl^ animals, *Prx1*-Cre homozygous male mice [33] were mated with homozygous *GATA6* ^fl/fl^ females [30]. The *Prx1*-Cre; *GATA6* ^fl/+^ male offspring of this mating were then backcrossed to *GATA6* ^fl/fl^ females to generate *Prx1*-Cre; *GATA6* ^fl/fl^ progeny. To generate *Prx1*-Cre; *GATA6* ^fl/fl^; *Gli1* ^−/−^ mice, we mated *Gli1* ^−/−^ and *GATA6* ^fl/fl^ animals to produce *Gli1* ^−/+^; *GATA6* ^fl/+^ animals, which were subsequently mated to *Prx1*-Cre males. The *Prx1*-Cre; *GATA6* ^fl/+^; *Gli1* ^−/+^ male offspring of this cross were mated to *GATA6* ^fl/fl^; *Gli1* ^−/−^ females, which had been generated by intercrossing *GATA6* ^fl/+^; *Gli1* ^−/+^ animals. *Prx1*-Cre; *GATA6* ^fl/fl^; *Shh* ^fl/fl^ mice were produced using a similar breeding scheme. *Prx1*-Cre; *ROSA26*-rtTA; *GATA6*-Tg (triple transgenic) animals were generated by mating *Prx1*-Cre; *ROSA26*-rtTA/rTA; *GATA6*-Tg males with C57Bl6 females. Doxycycline was administered to the pregnant females in their drinking water (2 mg/ml in 5% sucrose solution) starting at E4.5. Mildly affected triple transgenic animals were analyzed for this study.

### RT-qPCR and western blot on limb buds

Mouse E11.5 limb buds were dissected, cut in half as shown in Figure 1A and total cellular RNA was purified from each half using an RNeasy Plus Mini RNA isolation kit (Qiagen). RT-qPCR was performed using standard conditions [59]. qPCR analysis was conducted on a 7900HT real-time PCR machine (Applied Biosystems) using Fast SYBR Green Master Mix (Qiagen). Primers used in qPCR: GATA1- ATCAGCACTGGCCTACTACAGAG and GAGAGAAGAAAGGACTGGGAAAG, GATA2- CGCTCATCAAGCCCAAGC and ATTGTGCAGCTTGTAGTAGAGGCC, GATA3- CCTACTACGGAAACTCCGTCAGG, CAGGGCAGAGATCCGTGC, GATA4- TGGAAGACACCCCAATCTCG and TAGTGTCCCGTCCCATCTCG, GATA5- AACCGACCGCTAGTGAGGC and GCGTTGCACACTGGTTCG, GATA6- TACACAAGCGACCACCTCAG and CTATGTAGAGGCCGTCTTGACC, FOG1- GGAGACATGTCCAGGAGGAAAC and CCATGGCCTTGGCTTCTTC, FOG2- AGCCATTCAGACAAACCAGG and CATCTCTCTGAAACACTTCTAGCTCTC, Shh- CCGACATCATATTTAAGGATGAGG and CAAGGCATTTAACTTGTCTTTGC, Gli1- TCAAGGCCCAATACATGCTG and AGGACTTCCGACAGCCTTCA, Grem1- GGACCCACGGAAGTGACAG and GGCTCCTTGGGAACCTTTC. For Western analysis, mouse E11.5 limb buds were dissected as described above, proteins were separated by SDS-polyacrylamide gel electrophoresis, transferred to a membrane by Western blot, and the membranes probed with goat anti-Gli3 (AF3690, R&D systems) and mouse anti-alpha Tubulin (ab7750, Abcam) antibodies.

### Whole-mount in situ hybridization, cartilage and bone staining

Embryos were fixed overnight at 4°C in 4% paraformaldehyde. Whole-mount in situ hybridization was performed as described previously [60] with minor modifications. The following in situ probes were used: GATA6 exon 2 [29]; GATA6 3′UTR probe [28]; BMP2 and BMP4 [61]; Ptc1 [62]; FGF4 [63]; Shh, Hand2, Dhh, and Gli3 (Andy McMahon, University of Southern California); Gli1 (Alexandra Joyner, Sloan-Kettering Institute); Hoxd13 (Denis Duboule, Ecole Polytechnique Federale, Switzerland); FGF8 (Gail Martin, UCSF); BMP7 (Andrew Dudley, Northwestern University); Pax9, Grem1, Twist1, Alx4 (Cliff Tabin, Harvard Medical School), Ihh (Hank Kronenberg, MGH). Alcian Blue/Alizarin Red staining for cartilage and bone was performed as described previously [64].

### EMSA, luciferase assay, RNAi

In vitro translated GATA6 protein was produced using a TNT Quick Coupled Transcription/Translation System (Promega) according to manufacturer's protocol. EMSA was performed as described previously [65]. For luciferase assays, NIH3T3 cells were plated at 10^5^ cells/well into 6-well plates and transfected with indicated expression plasmids using Fugene 6 transfection reagent (Roche) according to manufacturer's protocol. To control for transfection efficiency, cells were co-transfected with SV40 driven renilla luciferase. Cells were lysed 48 h after transfection and luciferase reporter activities determined using the dual-luciferase reporter assay system (Promega). Gli3-targeting silencer select s66730 siRNA (Life Technologies) was transfected into NIH3T3 cells using Lipofectamine TNAiMAX transfection reagent (Invitrogen) according to the manufacturer's protocol.

### Chromatin IP from limb buds

ChIP was performed as described in [66] with minor modifications. Limb bud chromatin was sheared to approximately between 500–1000 bp prior to immunoprecipitation with anti-GATA6 antibody (Santa Cruz, sc-7244X). The following primers (as identified in Figures 5A and 6A) were used:

P1-forward: CATGGATGTAACTTGTCAGTTATATGGAG,

P1-reverse: ACATATGAGACCTAACAAGTGCAAGG,

P2-forward: GATTCAAAGAGACAGGGTACTCCC,

P2-reverse: AAGCCCAATCGGATGCC,

P3-forward: GGTCCTGGAGATCAAAAGCAG,

P3-reverse: GTTACAGGTGTGTACCACCATACC,

P4-forward: ATTAGTTGCACTGACCAGGTGG,

P4-reverse: CTATTGTGCTGTCATGTTGCTTG,

P5-forward: CCTCCATCTTAAAGAGAAGAGAGTAGG,

P5-reverse: GCAAAAATAATGAAAGAATCCAATG,

P6-forward: GGAATTGTTTTATATTCTCTTGTCTTAGG,

P6-reverse: ACCCAGCTACAGCAGCTTTC.

## Supporting Information

Figure S1 GATA6 is expressed in the anterior region of the limb bud mesenchyme. Whole mount in situ hybridization analysis of GATA6 expression in E10.5 (a, a', d, d'), E11.5 (b, b', e, e'), and E12.5 (c, c', f, f') WT (a–f) or *Prx1*-Cre; *GATA6* ^fl/fl^ (a'–f') forelimbs (a–c, a'–c') or hindlimbs (d–f, d'–f') performed with an exon2 mouse GATA6 probe [29]. Arrows point to the location of GATA6 expression. At least 6 limbs of each genotype were analyzed.
(PDF)

Figure S2 Loss of GATA6 in the forelimbs does not change expression of hedgehog responsive genes. (**A**) Alcian Blue/Alizarin Red staining of the forelimb of either a P0 control mouse (left) or a *Prx1*-Cre; *GATA6* ^fl/fl^ mouse (right) is displayed. (**B**) Whole mount in situ hybridization analysis of gene expression in mouse E11.5 forelimb buds from either control (a–e) or *Prx1-Cre; GATA6^fl/fl^* embryos (a'–e') with Shh (a, a'), Gli1 (b, b'), Ptc1 (c, c'), Grem1 (d, d'), and Hoxd13 (e, e') probes. At least 4 limbs of each genotype were analyzed for each in situ probe.
(PDF)

Figure S3 Expression of proteins recognized by anti-Gli3 antibody are decreased by RNAi knock-down of Gli3 RNA in NIH3T3 cells. Western blot analysis of Gli3FL and Gli3R levels in NIH3T3 cells transfected with increasing concentrations of either negative control siRNA (lanes 1–3) or siRNA against mouse Gli3 (lanes 4–6). siRNA targeting Gli3 specifically decreased expression of both immunoreactive proteins (lanes 4–6), indicating that anti-Gli3 antibody used in this study specifically recognizes Gli3 isoforms. Posterior (lane 7) and anterior (lane 8) halves of E11.5 hindlimb buds isolated from wild type mouse embryos are also shown. α–Tubulin is used as a loading control.
(PDF)

Figure S4 GATA6 does not bind to oligos from either the *Gli1* promoter or the *Shh* limb-specific enhancer, which contain mutated GATA6 binding sites. (**A**) GATA6 binding sites identified by EMSA analysis in the promoter of the mouse *Gli1* gene. Positions of GATA6 binding sites are indicated relative to the transcription start site of *Gli1*. Asterisk marks GATA6 binding site conserved between mouse and human genomes. (**B**) Electrophoretic mobility shift assay of in vitro translated GATA6 with either wild type GATA6-binding oligos (wt) or mutated oligos (m) from the *Gli1* promoter. Oligo 1 contains two GATA6 binding sites that were mutated separately (m1 and m2). Arrows point to the position of either the GATA6-oligo complexes or to the unbound labeled probes. (**C**) Sequence alignment of the conserved GATA6 binding site (indicated in bold) in the promoters of the human and mouse *Gli1* genes. (**D**) GATA6 binding sites identified by EMSA in the most conserved region (ShhE) of the mouse *Shh* limb bud enhancer ZRS. Position of GATA6 binding sites are indicated relative to the first HindIII site in the ZRS sequence [16]. Asterisks mark GATA6 binding sites conserved between mouse and human genomes. (**E**) EMSA of in vitro translated GATA6 with either wild type GATA6-binding oligos (wt) or mutated oligos (m) from the *Shh* limb bud enhancer. Oligo 7 contains two GATA6 binding sites that were mutated separately (m1 and m2). Arrows point to the position of either the GATA6-oligo complexes or to the unbound labeled probes. (**F**) Sequence alignment of the conserved GATA6 binding sites (indicated in bold) in the human and mouse Shh limb bud enhancer sequences. (**G**) EMSA of in vitro translated GATA6 with either an oligo containing the putative GATA6 binding site (WT) that is mutated in the Belgian 2 family [16] (lane 1), or a control GATA6 binding site (lane 2).
(PDF)
