# Supplementary figures and images for: GATA6 Is a Crucial Regulator of Shh in the Limb Bud
Source: PLoS Genet. 2014 Jan 9;10(1):e1004072. doi: 10.1371/journal.pgen.1004072 (PMC3886911; doi:10.1371/journal.pgen.1004072)

# GATA6

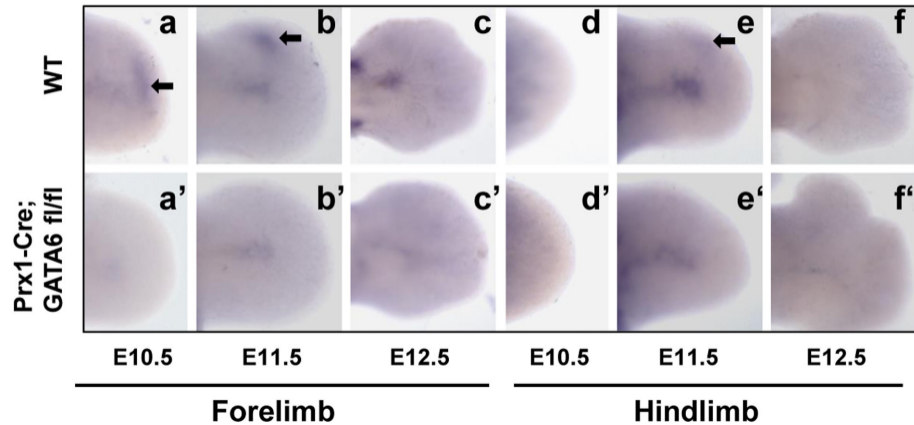

Supplement: Figure S1 — GATA6 is expressed in the anterior region of the limb bud mesenchyme. Whole mount in situ hybridization analysis of GATA6 expression in E10.5 (a, a', d, d'), E11.5 (b, b', e, e'), and E12.5 (c, c', f, f') WT (a–f) or Prx1-Cre; GATA6 fl/fl (a'–f') forelimbs (a–c, a'–c') or hindlimbs (d–f, d'–f') performed with an exon2 mouse GATA6 probe [29]. Arrows point to the location of GATA6 expression. At least 6 limbs of each genotype were analyzed. (PDF) [file pgen.1004072.s001.pdf]

**A**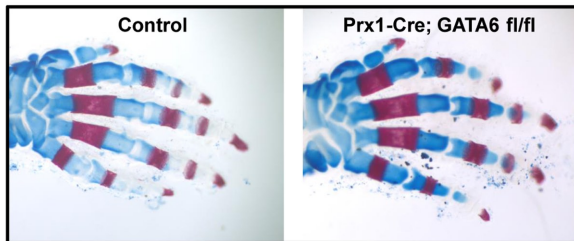**B**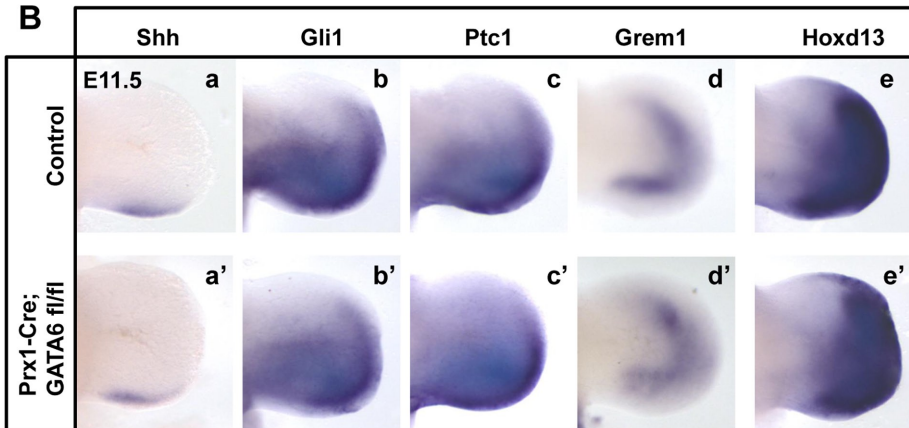

Supplement: Figure S2 — Loss of GATA6 in the forelimbs does not change expression of hedgehog responsive genes. (A) Alcian Blue/Alizarin Red staining of the forelimb of either a P0 control mouse (left) or a Prx1-Cre; GATA6 fl/fl mouse (right) is displayed. (B) Whole mount in situ hybridization analysis of gene expression in mouse E11.5 forelimb buds from either control (a–e) or Prx1-Cre; GATA6fl/fl embryos (a'–e') with Shh (a, a'), Gli1 (b, b'), Ptc1 (c, c'), Grem1 (d, d'), and Hoxd13 (e, e') probes. At least 4 limbs of each genotype were analyzed for each in situ probe. (PDF) [file pgen.1004072.s002.pdf]

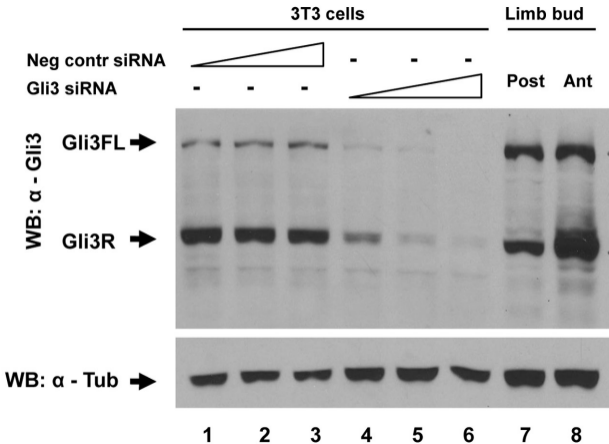

Supplement: Figure S3 — Expression of proteins recognized by anti-Gli3 antibody are decreased by RNAi knock-down of Gli3 RNA in NIH3T3 cells. Western blot analysis of Gli3FL and Gli3R levels in NIH3T3 cells transfected with increasing concentrations of either negative control siRNA (lanes 1–3) or siRNA against mouse Gli3 (lanes 4–6). siRNA targeting Gli3 specifically decreased expression of both immunoreactive proteins (lanes 4–6), indicating that anti-Gli3 antibody used in this study specifically recognizes Gli3 isoforms. Posterior (lane 7) and anterior (lane 8) halves of E11.5 hindlimb buds isolated from wild type mouse embryos are also shown. α–Tubulin is used as a loading control. (PDF) [file pgen.1004072.s003.pdf]
