# Supplementary material for: GATA6 Is a Crucial Regulator of Shh in the Limb Bud
Source: PLoS Genet. 2014 Jan 9;10(1):e1004072. doi: 10.1371/journal.pgen.1004072 (PMC3886911; doi:10.1371/journal.pgen.1004072)

A

Position	Sequence	Oligo #
-2331	ATGATTGC	1
-2323	CTGATTGG	1
-2127	TTGATATG	2
-1907	GAGATATA	3
-1425	CAGATAAG*	4
-973	GTGATAGG	5
-947	AAGATTGC	6

B

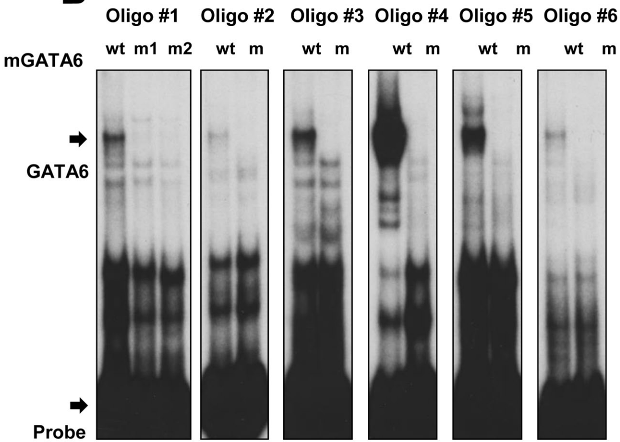

C

human CCAT**TATC**TC
mouse CCCT**TATC**TG

D

Position	Sequence	Oligo #
612	AAGATAGG	7
619	TTGATTTG*	7
1348	GAGATATG*	8

E

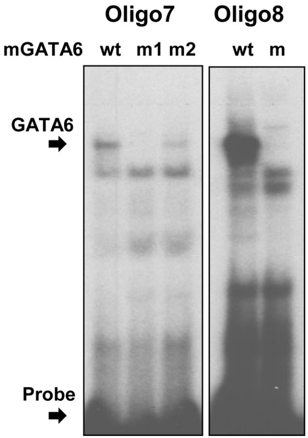

F

human TTT**GATT**TGA
mouse TTT**GATT**TGA

human CCA**TATC**TCA
mouse TCA**TATC**TCA

G

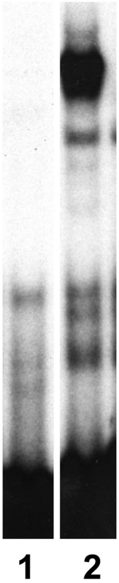

Supplement: Figure S4 — GATA6 does not bind to oligos from either the Gli1 promoter or the Shh limb-specific enhancer, which contain mutated GATA6 binding sites. (A) GATA6 binding sites identified by EMSA analysis in the promoter of the mouse Gli1 gene. Positions of GATA6 binding sites are indicated relative to the transcription start site of Gli1. Asterisk marks GATA6 binding site conserved between mouse and human genomes. (B) Electrophoretic mobility shift assay of in vitro translated GATA6 with either wild type GATA6-binding oligos (wt) or mutated oligos (m) from the Gli1 promoter. Oligo 1 contains two GATA6 binding sites that were mutated separately (m1 and m2). Arrows point to the position of either the GATA6-oligo complexes or to the unbound labeled probes. (C) Sequence alignment of the conserved GATA6 binding site (indicated in bold) in the promoters of the human and mouse Gli1 genes. (D) GATA6 binding sites identified by EMSA in the most conserved region (ShhE) of the mouse Shh limb bud enhancer ZRS. Position of GATA6 binding sites are indicated relative to the first HindIII site in the ZRS sequence [16]. Asterisks mark GATA6 binding sites conserved between mouse and human genomes. (E) EMSA of in vitro translated GATA6 with either wild type GATA6-binding oligos (wt) or mutated oligos (m) from the Shh limb bud enhancer. Oligo 7 contains two GATA6 binding sites that were mutated separately (m1 and m2). Arrows point to the position of either the GATA6-oligo complexes or to the unbound labeled probes. (F) Sequence alignment of the conserved GATA6 binding sites (indicated in bold) in the human and mouse Shh limb bud enhancer sequences. (G) EMSA of in vitro translated GATA6 with either an oligo containing the putative GATA6 binding site (WT) that is mutated in the Belgian 2 family [16] (lane 1), or a control GATA6 binding site (lane 2). (PDF) [file pgen.1004072.s004.pdf]
